# ﻿Molecular phylogeny and taxonomic position of *Macrobrachiumlanchesteri* (De Man, 1911), with descriptions of two new species from Thailand (Decapoda, Caridea, Palaemonidae)

**DOI:** 10.3897/zookeys.1190.113898

**Published:** 2024-01-29

**Authors:** Apisara Chaowvieng, Chirasak Sutcharit, Ratmanee Chanabun, Ruttapon Srisonchai, Ekgachai Jeratthitikul, Warut Siriwut

**Affiliations:** 1 Animal Systematics and Molecular Ecology Laboratory, Department of Biology, Faculty of Science, Mahidol University, Bangkok 10400, Thailand; 2 Animal Systematics Research Unit, Department of Biology, Faculty of Science, Chulalongkorn University, Bangkok 10330, Thailand; 3 Program in Animal Science, Faculty of Agricultural Technology, Sakon Nakhon Rajabhat University, Sakon Nakhon 47000, Thailand; 4 Biodiversity and Utilization Research Unit, Center of Excellence in Modern Agriculture, Sakon Nakhon Rajabhat University, Sakon Nakhon 47000, Thailand; 5 Department of Biology, Faculty of Science, Khon Kaen University, Khon Kaen 40002, Thailand

**Keywords:** Edible prawns, Lower Mekong Basin, morphological plasticity, new species

## Abstract

*Macrobrachiumlanchesteri* (De Man, 1911), a translucent freshwater prawn has a wide distribution range throughout mainland Southeast Asia. A high morphological variation and genetic divergence between different geographical *M.lanchesteri* populations in Thailand have peculiarly extended the uncertainty of species boundaries and blended confusingly with several *Macrobrachium* species. To clarify these circumstances, broad sample examinations of the morphological variation, including topotype specimens, and phylogenetic reconstruction based on the concatenated mitochondrial dataset (16s rRNA and COI genes) were performed. Broad morphological examination of *M.lanchesteri* has shown congruency with phylogenetic analyses by revealing prominent lineages of *M.lanchesteri* sensu stricto and two new sibling lineages with interspecific variation between 6.48–8.76% for COI and 3.06–4.23% for 16S. Descriptions of two new species, named herein as *M.panhai* Chaowvieng & Siriwut, **sp. nov.** and *M.rostrolevatus* Chaowvieng & Siriwut, **sp. nov.** are provided. Morphological investigation of rostral form suggested plasticity in *M.rostrolevatus* populations showing the morphological trait associated with their habitat preferences. Furthermore, phylogenetic positions of the three taxa affirmed the hidden diversity of Thai freshwater *Macrobrachium* fauna correlated with the river network in the Mekong and Chao Phraya basins, Thailand. The genetic data and distribution records obtained in this study may also assist future river conservation plans as well as the sustainable management of freshwater prawn diversity.

## ﻿Introduction

Palaemonid freshwater prawns of genus *Macrobrachium* Spence Bate, 1868 have shown high species richness comprising 271 species worldwide (WoRMS 2023). This genus has a broad geographical distribution and is commonly found in the Oriental Region of Asia (De Grave et al. 2008). Several *Macrobrachium* species demonstrate economic impacts, serving as protein resources and for utilisation in ornamental fish aquaculture (Cai et al. 2004; Wowor et al. 2004). According to its remarkable species richness and diversifications of aquatic and terrestrial invertebrate faunas in Indochina, the intensive fauna exploration and historical biogeography using both morphology and genetics were reinvestigated systematically in several taxa such as river prawns, bivalves, land snails and millipedes (De Bruyn et al. 2014; Pholyotha et al. 2021; Jeratthitikul et al. 2022; Likhitrakarn et al. 2023). Indochinese *Macrobrachium* prawns have gained attention recently, especially in the context of taxonomy and systematics (Cai et al. 2004; Wowor et al. 2004; Hanamura et al. 2011). Several molecular taxonomic studies have been verified nominal species and consequently supplemented the taxonomic account of some cryptic *Macrobrachium* prawns (Mar et al. 2018; Siriwut et al. 2020; Jurniati et al. 2021; Saengphan et al. 2021). Additionally, the DNA barcoding and molecular delimitation methods were implemented to clarify the taxonomic boundaries of several *Macrobrachium* species. Moreover, the phylogenetic positions of several species have been addressed some morphological complexity groups based on barcode gap distance threshold (Siriwut et al. 2021).

Currently, 34 species have been documented in Thailand (Cai et al. 2004; Cai and Vidthayanon 2016; Saengphan et al. 2018, 2019, 2020, 2021; Siriwut et al. 2020, 2021). Two major river basins, the Chao Phraya and the Greater Mekong, have been discussed as being significant hotspots for native *Macrobrachium* faunal diversity (Cai and Ng 2002; Hanamura et al. 2011). Some Thai *Macrobrachium* species have been reported to show narrow distribution within these basins, such as *M.chainatense* Saengphan, Panijpan, Senapin, Laosinchai, Ruenwongsa, Suksomnit & Phiwsaiya, 2019 which was only found in Central Thailand, and *M.spelaeus* Cai & Vidthayanon, 2016 that live in stygobiotic habitats. Contrastingly, some widespread species have also been documented about their distribution occupancy crossed inland basins and some insular territory of Southeast Asia, such as *M.sintangense* (De Man, 1898), and *M.dienbienphuense* Dang & Nguyen, 1972 (Cai et al. 2004; Wowor et al. 2004; Hanamura et al. 2011). For this reason, freshwater faunas in Thailand and neighbouring countries are capable linkage in terms of species composition, reaching an occurrence data of coexistence and cryptic species according to the connection of the river network (Hanamura et al. 2011; Siriwut et al. 2020).

A small translucent and common *M.lanchesteri* (De Man, 1911) dominantly occupies all river basins throughout mainland Southeast Asia with scattered distribution records from Malaysia, Singapore, Indonesia; it has even expanded northward to South China (Wowor and Choy 2001; Cai and Ng 2002; Cai et al. 2004). This species was originally found in southern Thailand and was diagnosed as having a straight and short rostrum not exceeding the scaphocerite and slender, thin second pereiopods (Kemp 1918; Holthuis 1950). The lectotype designation and morphological study of *M.lanchesteri* by Chong and Khoo (1988) advocated diagnostic character variation, particularly on rostral structure and body size variation in male regarding sexual dimorphism. Additionally, *M.lanchesteri* was mentioned with an argument on taxonomic boundary with some other congeners such as *M.peguense* (Tiwari, 1952), *M.kistnense* (Tiwari, 1952), and *M.tiwarii* Jalihal, Shenoy & Sankolli, 1988. Moreover, *M.lanchesteri* also blended confusingly with the juveniles of several species such as *M.idae* (Heller, 1862) and *M.lar* (Fabricius, 1798) (Lanchester 1902; Kamita 1966).

Previous phylogenetic and population genetic studies of *M.lanchesteri* in Thailand have also detected high genetic diversity, both between and within populations (Reingchai et al. 2009; Khanarnpai et al. 2019; Siriwut et al. 2021). Moreover, the possible existence of cryptic species within several *Macrobrachium* species in Thailand under traditional morphological discrimination criteria was reported, including *M.lanchesteri*, based on DNA barcode delimitation thresholds (Siriwut et al. 2021). The lack of intensive collection from different river basins impeded comprehensive genetic and morphological information that would contribute to taxonomic boundary clarification and phylogenetic relationships of *M.lanchesteri* and other native species in this area. To elucidate the taxonomic confusion of several coexistent translucent *Macrobrachium* prawns, the integration of traditional morphological identification and molecular analysis could be investigated concurrently. Therefore, this study aimed to clarify the taxonomic boundaries of *M.lanchesteri* in Thailand by broad-scale sampling and reconstruct the phylogenetic relationships with various related translucent species based on COI gene and 16S rRNA markers, which have been used extensively to investigate the phylogenetic relationships between crustaceans (Costa et al. 2007; Pileggi and Mantelatto 2010; Castelin et al. 2017; Jamaluddin et al. 2019; Rossi et al. 2020). This study will contribute to elucidate the taxonomic status of *M.lanchesteri* s. str.and its closely related species as well as assist economical freshwater prawn management in the future.

## ﻿Materials and methods

### ﻿Sample collection and preparation

Prawn specimens were collected from various freshwater basins in Thailand. Live specimens were photographed to document body coloration using a Nikon D5300 camera with a micro-Nikkor 105 mm f/2.8 IF-ED Macro Lens. Prawns were gradually euthanised following the protocols approved by the Mahidol University-Institute Animal Care and Use Committee (MU-IACUC) under approval number MUSC66-026-656. Specimens were preserved in 95% ethanol and stored into a container for further morphological examination and molecular analysis. Voucher specimens were deposited at the Chulalongkorn University Museum of Zoology, Bangkok, Thailand (**CUMZ**) and Mahidol University Museum of Natural History, Department of Biology, Faculty of Science, Mahidol University, Thailand (**MUMNH**). Traditional identifications were carried out based on previous taxonomic studies of *Macrobrachium* species: Lanchester (1902), Holthuis (1950), Chong and Khoo (1988), Cai and Ng (2002), Cai et al. (2004), Wowor et al. (2004), and Hanamura et al. (2011). The morphological variation of prawn specimens was observed and illustrated under a stereomicroscope. A list of abbreviations used in the descriptions is given as follows: **Fin** (finger), **Pal** (palm), **Car** (carpus), **Mer** (merus), **Che** (chela), **Dac** (dactylus), **Pro** (propodus), **cl** (carapace length), **rl** (rostrum length). All morphological characters were measured using Dinocapture software v. 2.0 and reported in millimetres.

### ﻿DNA extraction and PCR protocol

All prawn specimens used for molecular analysis in this study are listed in Table 1. Genomic DNA was extracted from pleonal muscle tissue by using DNA extraction kits (NucleoSpin Tissue kit: MACHEREY-NAGEL). Genomic DNA quality was evaluated and visualised by gel electrophoresis and a UV illuminator. Two mitochondrial genes, 16S rRNA and cytochrome c oxidase subunit I (COI), were amplified. Three sets of primer such as 16Sa-L (5’ CGC CTG TTT ATC AAA AAC AT 3’) and 16Sbr-H2 (5’ CTC CGG TTT GAA CTC AGA TCA 3’) following Palumbi (1996) for 16S gene, LCO1490 (5’GGT CAA CAA ATC ATA AAG ATA TTG G 3’; Folmer et al. (1994), MacroNancy (5’ GCG GGT AGR ATT AAR ATR TAT ACT TC 3’; Siriwut et al. (2020), HCOoutout (5’ GTA AAT ATA TGR TGD GCTC 3’; Schulmeister et al. (2002) for COI were used in this study. PCR was performed using T100^TM^ thermal cycler (BIO-RAD) with a gradient temperature function. The PCR profile consisted of the following steps: 94 °C for 5 min as an initial step followed by 34 cycles 94 °C for 30 sec for denaturing, 45–49 °C for 40 sec, 72 °C for 15 sec for extension, and final extension at 72 °C for 10 min. PCR products were run by 1% agarose gel electrophoresis stained with SYBR Safe illuminant (Invitrogen, USA). The purified products were sent for sequencing by a commercial company (Macrogen and Bioneer, Korea) using an Applied Biosystems automatic sequencer.

**Table 1.** Locality and GenBank accession numbers of specimens used in phylogenetic analyses.

Taxa	Voucher IDs	Localities	Coordinates	GenBank accession no.		References
				COI	16S	
*M.lanchesteri* (De Man, 1911)	MUMNH_MP00221.1- M421	Yom, Pong, Phayao	19°06'24.3"N, 100°15'58.5"E	OR575100	OR578680	This study
	MUMNH_MP00230.1-M420	Pai, Mueang Mae Hong Son, Mae Hong Son	19°19'36.89"N, 97°56'34.78"E	OR575099	OR578679	This study
	MUMNH_MP00350-M400	Nam Lao, Mae Taeng, Chiang Mai	19°12'16.4"N, 98°40'51.3"E	OR575091	OR578671	This study
	MUMNH_ MP00222.1-M379	Mae Mang, Bo Kluea, Nan	19°08'12.6"N, 101°09'03.1"E	OR575081	OR578661	This study
	MUMNH_MP00245.1-M437	Khlong Khlung, Wang Sai, Kamphaeng Phet	16°11'51.7"N, 99°36'53.0"E	OR575108	OR578688	This study
	MUMNH_MP00259.1-M443	Klong Un, Phu Phan, Sakon Nakhon	17°00'22.1"N, 103°54'50.5"E	OR575112	OR578692	This study
	MUMNH_MP00259.1-M433	Klong Un, Khok Phu, Phu Phan, Sakon Nakhon	17°00'22.1"N, 103°54'50.5"E	OR575106	OR578686	This study
	MUMNH_MP00262.1-M442	Huai Sathot, Huai Phueng, Kalasin	16°41'32.6"N, 103°51'20.1"E	OR575111	OR578691	This study
	MUMNH_MP00264.1-M399	Chi River, Mueang Maha Sarakham, Maha Sarakham	16°13'01.1"N, 103°16'44.1"E	OR575090	OR578670	This study
	MUMNH_MP00274.1-M435	Lam Takhong, Pak Chong, Nakhon Ratchasima	14°33'00.7"N, 101°27'34.1"E	OR575107	OR578687	This study
	MUMNH_MP00278.1-M452	Nong Prue Mai Kaeo, Plaeng Yao, Chachoengsao	13°33'31.5"N, 101°17'44.0"E	OR575118	OR578698	This study
	MUMNH_MP00293.1-M412	Yang Chum, Tha Yang, Phetchaburi	12°46'55.7"N, 99°40'43.65"E	OR575098	OR578678	This study
	MUMNH_MP00300.1-M398	Pak Nam, Mueang Krabi, Krabi	8°04'50.7"N, 98°55'07.3"E	OR575089	OR578669	This study
	MUMNH_MP00301.1-M422	Klong Na Thap, Chana, Songkhla	7°01'20.1"N, 100°43'51.5"E	OR575101	OR578681	This study
	CUMZ_MP00089-M078	Sathing Phra, Songkhla	7°25'01.4"N, 100°25'04.0"E	MW845498	OR578643	Siriwut et al. 2021
	CUMZ_MP00090-M079	Sathing Phra, Songkhla	7°25'01.4"N, 100°25'04.0"E	MW845497	OR578644	Siriwut et al. 2021
	CUMZ_MP00093-M082	La-un, Ranong	10°06'37.1"N, 98°45'32.5"E	MW845495	OR578645	Siriwut et al. 2021
*M.panhai* sp. nov.	MUMNH_MP00307.1-M441	Klong Tron, Thong Sang Khan, Uttaradit	17°35'39.1"N, 100°29'10.3"E	OR575110	OR578690	This study
	MUMNH_MP00351-M447	Klong Hi, Seka, Bueng Kan	17°54'17.9"N, 103°57'12.0"E	OR575115	OR578695	This study
	MUMNH_MP00309.1-M404	Nam Suai, Mueang Nong Khai, Nong Khai	17°45'01.1"N, 102°51'00.5"E	OR575092	OR578672	This study
	MUMNH_MP00310.1-M425	Mekong River, Khong Chiam, Ubon Ratchathani	15°19'10.3"N, 105°29'53.2"E	OR575103	OR578683	This study
	MUMNH_MP00313.1-M439	Ang Kep Nam Nam Khuen Nam Lang, Wang Pong, Phetchabun	16°25'19.8"N, 100°48'48.2"E	OR575109	OR578689	This study
	CUMZ_MP00302-M382	Sri Nakarin Dam, Si Sawat, Kanchanaburi	14°24'12.1"N, 99°07'24.7"E	OR575082	OR578662	This study
	MUMNH_MP00320.1-M405	Huai Raeng, Bo Rai, Trat	12°23'48.4"N, 102°39'15.1"E	OR575093	OR578673	This study
	CUMZ_MP00020-M003	Nam Pat, Uttaradit	17°43'47.0"N, 100°41'24.3"E	MW845582	OR578642	Siriwut et al. 2021
	CUMZ_MP00146-M147	Klaeng, Rayong	12°47'05.7"N, 101°40'59.6"E	MW845580	OR578651	Siriwut et al. 2021
*M.rostrolevatus* sp. nov.	CUMZ_MP00323-M368	Bueng Khong Long, Bueng Kan	17°59'59.1"N, 104°01'06.9"E	OR575076	OR578656	This study
	MUMNH_MP00324.1-M387	Bueng Khong Long, Bueng Kan	17°59'59.1"N, 104°01'06.9"E	OR575083	OR578663	This study
	MUMNH_MP00324.2-M408	Bueng Khong Long, Bueng Kan	17°59'59.1"N, 104°01'06.9"E	OR575095	OR578675	This study
	MUMNH_MP00325.1-M392	Nam Suai, Mueang Nong Khai, Nong Khai	17°45'01.1"N, 102°51'00.5"E	OR575085	OR578665	This study
	MUMNH_MP00326.1-M388	Si Charoen, Phen, Udon Thani	17°42'47.7"N, 102°50'57.4"E	OR575084	OR578664	This study
	MUMNH_MP00352-M407	Tha Rae, Mueang Sakon Nakhon, Sakon Nakhon	17°15'08.5"N, 104°09'32.0"E	OR575094	OR578674	This study
	MUMNH_MP00330.1-M424	Nam Chan, Akat Amnuai, Sakon Nakhon	17°35'46.1"N, 104°00'21.6"E	OR575102	OR578682	This study
	MUMNH_MP00334.1-M367	Bueng Aram, Yang Talat, Kalasin	16°24'21.8"N, 103°20'26.4"E	OR575075	OR578655	This study
*M.rostrolevatus* sp. nov.	MUMNH_MP00334.2-M409	Bueng Aram, Yang Talat, Kalasin	16°24'21.8"N, 103°20'26.4"E	OR575096	OR578676	This study
	MUMNH_MP00334.3-M432	Bueng Aram, Yang Talat, Kalasin	16°24'21.8"N, 103°20'26.4"E	OR575105	OR578685	This study
	MUMNH_MP00353-M364	Suk San, Khun Han, Si Sa Ket	14°35'27.7"N, 104°29'29.3"E	OR575072	OR578652	This study
	MUMNH_MP00340.1-M444	Huai Khayung, Kantharalak, Si Sa Ket	14°34'42.1"N, 104°38'48.1"E	OR575113	OR578693	This study
	MUMNH_MP00343.1-M371	Mun River, Tha Tum, Surin	15°17'38.5"N, 103°30'42.4"E	OR575077	OR578657	This study
	MUMNH_MP00348.1-M445	Sawai Riang, Non Sung, Nakhon Ratchasima	15°16'13.3"N, 102°22'37.0"E	OR575114	OR578694	This study
	MUMNH_MP00341.1-M448	Klong Thap Than, Rattanaburi, Surin	15°16'55.4"N, 103°58'38.1"E	OR575116	OR578696	This study
	MUMNH_MP00354-M450	Tha Yang, Phu Kradueng, Loei	16°53'38.3"N, 101°52'53.1"E	OR575117	OR578697	This study
	MUMNH_ MP00346.1-M411	Sathaet, Mueang Yang, Nakhon Ratchasima	15°27'35.2"N, 102°59'46.6"E	OR575097	OR578677	This study
	CUMZ_MP00096-M085	Mueang, Mahasarakam	16°11'01.4"N, 103°27'24.4"E	MW845577	OR578646	Siriwut et al. 2021
	CUMZ_MP00097-M086	Udonthani	17°19'02.5"N, 102°35'53.0"E	MW845578	OR578647	Siriwut et al. 2021
*M.rosenbergii* (De Man, 1879)	MUMNH_MP00355-M377	Pak Chan, Kra Buri, Ranong	10°31'38.0"N, 98°50'01.8"E	OR575080	OR578660	This study
	MUMNH_MP00356-M394	Pak Chan, Kra Buri, Ranong	10°31'38.0"N, 98°50'01.8"E	OR575087	OR578667	This study
	CUMZ_MP00100-M094	Mueang, Maha Sarakham	16°11'01.4"N, 103°27'24.4"E	MW845593	OR578648	Siriwut et al. 2021
	CUMZ_MP00118-M115	Mueang, Ranong	9°53'13.5"N, 98°38'01.2"E	MW845595	OR578650	Siriwut et al. 2021
*M.sintangense* (De Man, 1898)	MUMNH_MP00357-M374	Huai Yang, Ta Phraya, Sa Kaeo	14°00'44.4"N, 102°38'39.9"E	OR575078	OR578658	This study
	MUMNH_MP00358-M393	Huai Yang, Ta Phraya, Sa Kaeo	14°00'44.4"N, 102°38'39.9"E	OR575086	OR578666	This study
	MUMNH_MP00359-M366	Khwae Noi, Mueang Kanchanaburi, Kanchanaburi	13°58'18.4"N, 99°18'25.9"E	OR575074	OR578654	This study
	MUMNH_MP00360-M430	Kui Buri, Prachuap Khiri Khan	12°05'29.7"N, 99°48'18.1"E	OR575104	OR578684	This study
	MUMNH_MP00361-M365	Tha Di, Nakhon Si Thammarat	8°23'28.5"N, 99°52'27.4"E	OR575073	OR578653	This study
*M.villosimanus* (Tiwari, 1949)	MUMNH_MP00362-M376	Chan Thi, Mueang Trat, Trat	12°15'55.0"N, 102°36'08.8"E	OR575079	OR578659	This study
	MUMNH_MP00363-M396	Trang, Kantang, Trang	7°26'02.9"N, 99°30'54.9"E	OR575088	OR578668	This study
	CUMZ_MP00116-M113	La-ngu, Satun	6°54'22.3"N, 99°48'42.2"E	MW845638	OR578649	Siriwut et al. 2021

### ﻿Phylogenetic analyses

**Figure 1.**
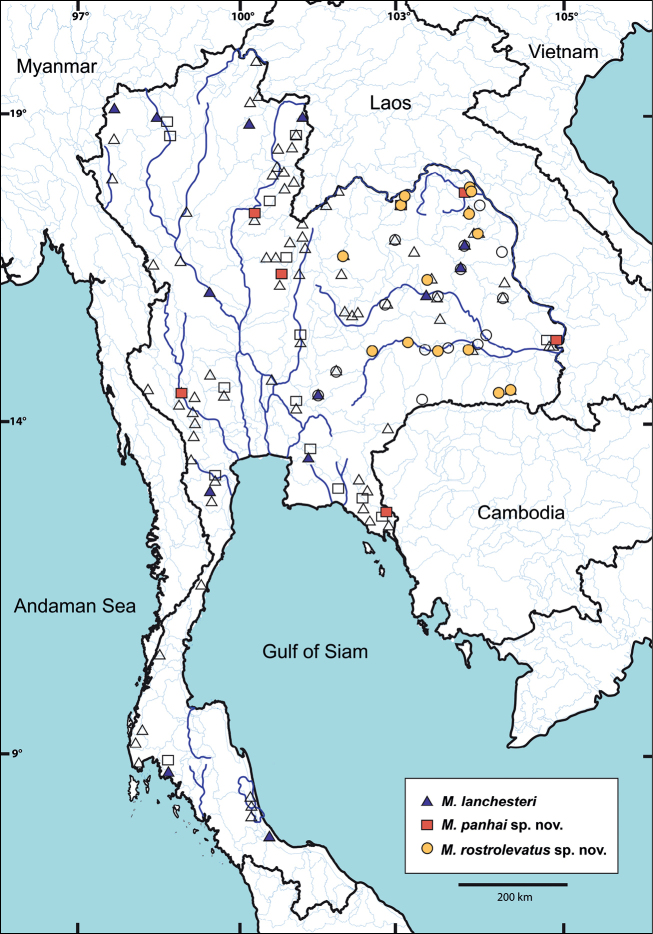
Distribution map of three *Macrobrachium* species in Thailand. A colour symbol indicates the locality of specimen used in phylogenetic analyses. A transparent symbol indicates the locality of specimen examined based on morphology. Equivalent symbols, whether coloured or not, indicate the same species.

Sequences were aligned and corrected using the ClustalW algorithm in MEGA 11 (Tamura et al. 2021). All sequences have been registered and deposited in GenBank database under accession numbers OR575072–OR575118 for COI and OR578642–OR578698 for 16S (Table 1). The voucher specimen locality of each species used in molecular analysis is illustrated in Fig. 1. The DNA dataset for phylogenetic analyses was assembled including ten deposited COI sequences of *Macrobrachium* species in GenBank database. To depict the clade of *M.lanchesteri* sensu De Man (1911), topotype sequences were selected as representative indicators. *Macrobrachiumvillosimanus* (Tiwari, 1949) was used as the rooting outgroup.

Phylogenetic trees were constructed using maximum likelihood (ML) and Bayesian inference (BI) methods throughout the online CIPRES Science Gateway server (Miller et al. 2010). The concatenated dataset of two markers with the partitioned file for nucleotide substitution model fit was prepared in Kakusan 4 (Tanabe 2007). ML tree was visualised in RAxML v. 8.2.12. (Stamatakis 2014). The GTR+G model was set as the model for all gene partitions with 1,000 bootstrap replicates performed to verify tree topology and clade support. BI tree was estimated using MrBayes v. 3.2.7 (Ronquist et al. 2012). Markov chain Monte Carlo (MCMC) was configured as 10,000,000 generations of the sampling process; the first 25% of obtained trees were discarded as burn-in. Finalised trees were estimated for the consensus tree topology. The annotation and illustration of clade and branch length were performed in Figtree (Rambaut 2010). Node posterior probabilities of 0.95 were considered statistically significant for BI, and bootstrap support values greater than 70 were considered highly supported for ML (Huelsenbeck and Hillis 1993; Larget and Simon 1999). Pairwise genetic distance of intra and interspecific of each gene dataset was calculated using the p-distance method in MEGA 11 (Tamura et al. 2021).

## ﻿Results

### ﻿Molecular phylogeny and genetic divergence

Forty-seven sequences of partial COI and 57 sequences of partial 16S genes were successfully amplified and obtained (Table 1). COI sequence contained 627 bp with 417 bp of conserved sites, 210 bp of variable sites and 202 of parsimony informative sites. 16S sequence contained 554 bp with 373 bp of conserved sites, 181 bp of variable sites and 154 bp of parsimony informative sites. The proportional range of genetic variations in *M.lanchesteri* species complex and other *Macrobrachium* species were revealed by p-distance. Inter and intraspecific variations ranged from 15.12–20.68% for COI, 8.6–16.18% for 16S and 0.9–5.79% for COI and 1.08–3.19% for 16S, respectively.

**Figure 2.**
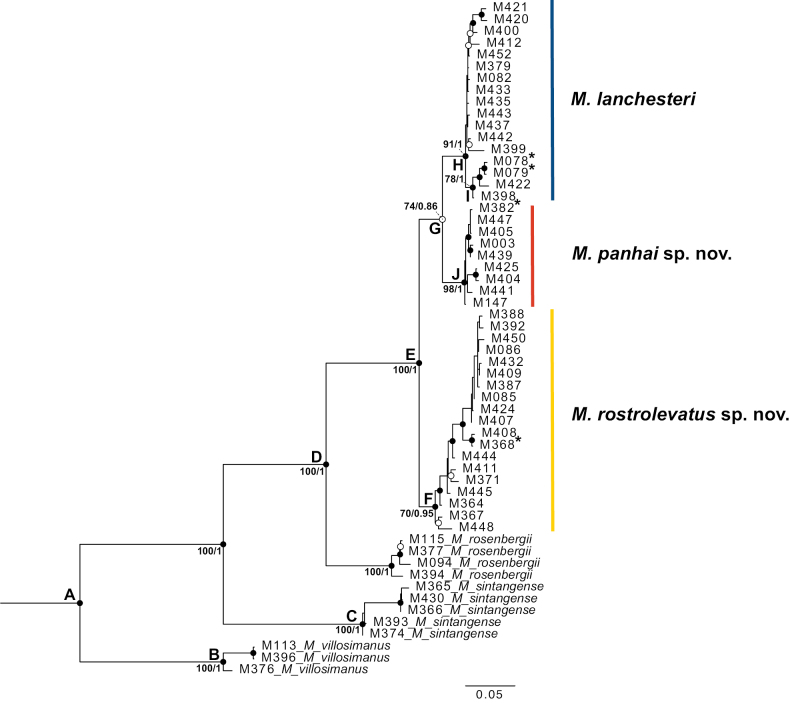
Phylogenetic tree based on a concatenation of COI and 16S genes. Nodes of a phylogenetic tree marked with a black circle indicate statistical support from both ML and BI (≥ 70 bootstrap values and ≥ 0.95 posterior probability scores). A white circle indicates statistical support for either ML or BI. An asterisk indicates the topotype in *M.lanchesteri* and holotype in the new species.

Both ML and BI trees based on 1,181 bp concatenated dataset of the COI and 16S gene fragments revealed the six *Macrobrachium* species as monophyletic groups with strong statistical support values (Fig. 2). Clade C comprised all *M.sintangense* sequences. Phylogenetic tree also showed that *M.rosenbergii* (De Man, 1879) is closely related to *M.lanchesteri* species complex clade, forming clade D. The genetic distance between *M.rosenbergii* and *M.lanchesteri* species complex clade was 15.12% for COI and 8.6% for 16S. In the clade E, *Macrobrachiumlanchesteri* species complex was divided into three monophyletic groups with high statistical supports for both ML (100) and BI (1). The interspecific variation ranged from 6.48–8.76% for COI and 3.06–4.23% for 16S. The intraspecific variation also ranged from 0.92–2.27% for COI and 0.7–2.23% for 16S. In the results of this study, clade H was shown as *M.lanchesteri* based on the topotype sequences assembled. The monophyletic group of *M.lanchesteri* s. str. herein represents two subclades, lower Isthmus of Kra (Clade I) and upper Isthmus of Kra populations. *Macrobrachiumpanhai* sp. nov. (Clade J) was nested as a sister clade of *M.lanchesteri* s. str. with sufficient support in ML (74), but partial support in BI (86). *Macrobrachiumrostrolevatus* sp. nov. (Clade F) was separated from congeneric members of *M.lanchesteri* species complex and all samples in this clade were strictly distributed inside freshwater basins on the Khorat Plateau, i.e. the Mun, Chi and Songkhram Rivers.

### ﻿Taxonomic account

#### ﻿ Macrobrachium lanchesteri

Taxon classificationAnimaliaDecapodaPalaemonidae

(De Man, 1911)

40A14955-8481-5F94-B6AE-42D54B8FCE84

Figs 3A, B , 4

Palaemon paucidens Lanchester, 1902: 568, pl. 33, fig. 4. Type locality: Singora [Songkhla Province, Thailand]. [Not De Haan (1844) and Hilgendorf (1893)].
Palaemon (Eupalaemon) lanchesteri De Man, 1911: 264. Replacement Name.
Palaemon lanchesteri : Kemp 1918: 257.
Macrobrachium lanchesteri : Suvatti 1937: 49; Suvatti 1967; 139; Holthuis 1950: 139; Holthuis 1980: 96; Johnson 1961: 56; Naiyanetr 1980: 17; Naiyanetr 1992: 17; Naiyanetr 1998: 32; Chong and Khoo 1988: 196; Cai and Ng. 2002: 77; Cai et al. 2004: 586; Wowor et al. 2004: 349; Hanamura et al. 2011: 13.
Macrobrachium lar : Kamita 1966: 138.
Cryphiops (Macrobrachium) lanchesteri : Johnson 1968: 233.
Macrobrachium cf. lanchesteri : Ng 1994: 75, fig. 2.

##### Material examined.

***Topotypes*: Songkhla** • 11 ♀♀, 3 ♂♂, Wat Pho Klang, Khu Khut, Sathing Phra; 7°25'26.2"N, 100°25'05.8"E; MUMNH MP00216. • 25 ♀♀, 3 ♂♂, Laem Wang, Khu Khut, Sathing Phra; 7°27'12.3"N, 100°25'12.9"E; MUMNH MP00217. • 5 ♀♀, 3 ♂♂, Bang Khiat, Singhanakhon; 7°20'19.2"N, 100°25'36.8"E; MUMNH MP00218.

##### Additional material.

**Chiang Rai** • 12 ♀♀, 16 ♂♂, Chiang Khong Street Market, Wiang, Chiang Khong; 20°15'57.7"N, 100°24'21.7"E; MUMNH MP00219. **Chiang Mai** • 1 ♀, Nam Lao, Pa Pae, Mae Taeng; 19°12'16.4"N, 98°40'51.3"E; MUMNH MP00350. **Phayao** • 5 ♀♀, 1 ovigerous, 4 ♂♂, Wean, Ban Pa Suk, Chiang Kham; 19°30'04.7"N, 100°16'32.9"E; MUMNH MP00220. • 17 ♀♀, 14 ♂♂, Yom, Ban Duea, Pong; 19°06'24.3"N, 100°15'58.5"E; MUMNH MP00221. **Nan** • 4 ♀♀, Mae Mang, Bo Kluea Tai, Bo Kluea; 19°08'12.6"N, 101°09'03.1"E; MUMNH MP00222. • 10 ♀♀, 1 ovigerous, 8 ♂♂, Ban Hua Fai, Sisaket, Na Noi; 18°19'33.4"N, 100°43'25.6"E; MUMNH MP00223. • 1 ovigerous, Huai Pa Sak, Sisaket, Na Noi; 18°19'33.4"N, 100°43'25.6"E; MUMNH MP00224. • 1 ♀, 1 ♂, Ban Huai Lao, Chiang Khong, Na Noi; 18°18'42.3"N, 100°54'18.2"E; MUMNH MP00225. • 2 ♀♀, 1 ovigerous, 4 ♂♂, Huai Hin, Sathan, Na Noi; 18°14'21.8"N, 100°41'57.7"E; MUMNH MP00226. • 2 ♀♀, Ban Na Bua, Nong Daeng, Mae Charim; 18°45'12.8"N, 101°00'39.7"E; MUMNH MP00227. • 3 ♀♀, 1 ♂, Nan River, Tan Chum, Wiang Sa; 19°00'59.5"N, 100°46'47.3"E; MUMNH MP00228. **Lampang** • 1 ♀, 2 ♂♂, Ban Mae Pa, Thoen; 17°38'17.9"N, 99°15'39.6"E; MUMNH MP00229. **Mae Hong Son** • 3 ♀♀, 8 ♂♂, Pai, Pang Mu, Mueang Mae Hong Son; 19°19'36.9"N, 97°56'34.8"E; MUMNH MP00230. • 2 ♀♀, 2 ♂♂, Yuam, Mae Sariang; 18°09'26.0"N, 97°55'37.8"E; MUMNH MP00231. • 8 ♀♀, 4 ♂♂, Nong Pong Sila, Khun Yuam; 18°50'26.4"N, 97°56'21.3"E; MUMNH MP00232. **Uttaradit** • 3 ♀♀, 5 ♂♂, Huai Nam Muet, Phak Khuang, Thong Sang Khan; 17°28'21.5"N, 100°22'25.6"E; MUMNH MP00233. • 4 ♀♀, 1 ovigerous, 3 ♂♂, Klong Tron, Ban Bueng Pra Kot, Thong Sang Khan; 17°35'39.1"N, 100°29'10.3"E; MUMNH MP00234. • 4 ♀♀, 15 ♂♂, Nam Pat, Fak Tha; 18°00'04.4"N, 100°52'42.7"E; MUMNH MP00235. • 2 ♀♀, Ban Rai Phana Wan, Muang Chet Ton, Ban Khok; 18°08'48.7"N, 101°02'11.0"E; MUMNH MP00236. **Tak** • 2 ♀♀, 2 ♂♂, Ban Klong Haui Sai, Nong Bua Tai, Mueang Tak; 16°46'24.2"N, 99°06'45.0"E; MUMNH MP00237. • 1 ♀, Klong Mae Sot, Phra That Pha Daeng, Mae Sot; 16°42'24.7"N, 98°36'52.1"E; MUMNH MP00238. **Phitsanulok** • 13 ♀♀, 7 ♂♂, Khek, Kaeng Sopha, Wang Thong; 16°53'15.4"N, 100°39'13.3"E; MUMNH MP00239. • 7 ♀♀, 3 ♂♂, Ban Bo, Wang Nok Aen, Wang Thong; 16°51'13.6"N, 100°36'43.2"E; MUMNH MP00240. **Phetchabun** • 16 ♀♀, 5 ♂♂, Ban Pho Ngam, Tha Phon, Mueang Phetchabun; 16°35'29.5"N, 101°07'41.6"E; MUMNH MP00241. • 4 ♀♀, 2 ♂♂, Si Thep Historical Park, Si Thep;15°28'19.7"N, 101°08'48.2"E; MUMNH MP00242. • 1 ovigerous, 1 ♂, Nam Khuen Nam Lang, Wang Hin, Wang Pong; 16°24'20.6"N, 100°47'56.5"E; MUMNH MP00243. • 5 ♀♀, 1 ♂, Klong Nam Phung, Hin Hao, Lom Kao; 16°58'47.8"N, 101°12'51.5"E; MUMNH MP00244. **Kamphaeng Phet** • 4 ♀♀, 2♂♂, Khlong Khlung, Wang Sai; 16°11'51.7"N, 99°36'53.0"E; MUMNH MP00245. **Nong Khai** • 4 ♀♀, 4 ovigerous, 2 ♂♂, Nam Suai, Song Hong, Mueang Nong Khai; 17°45'01.1"N, 102°51'00.5"E; MUMNH MP00246. **Loei** • 5 ovigerous, 1 ♂, Mekong River, Hat Bia, Pak Chom; 18°03'39.4"N, 101°47'51.8"E; MUMNH MP00247. • 3 ♀♀, 1 ovigerous, 8 ♂♂, Hueang, Na Chan, Chaing Khan; 17°47'13.7"N, 101°34'31.1"E; MUMNH MP00248. • 2 ♀♀, 1 ovigerous, 4 ♂♂, Klong Nam Man, Na Ho, Dan Sai; 17°19'35.6"N, 101°08'54.5"E; MUMNH MP00249. • 4 ♀♀, Klong Khok Khamin, Khok Khamin, Wang Saphung; 17°10'24.7"N, 101°50'52.1"E; MUMNH MP00250. • 4 ♀♀, 3 ovigerous, 1 ♂, Ban Wang Kum, Dan Sai; 17°07'27.6"N, 101°10'40.7"E; MUMNH MP00251. • 4 ♀♀, 2 ♂♂, Ban Tha Yang, Phu Kradueng; 16°53'47.6"N, 101°53'18.3"E; MUMNH MP00252. **Udon Thani** • 1 ♀, Klong Nam Khong, Thap Kung, Nong Saeng; 17°10'01.5"N, 102°46'03.2"E; MUMNH MP00253. • 1 ♀, 2 ovigerous, 2 ♂♂, Huai Yang, Nong Ya Sai, Wang Sam Mo; 16°59'19.2"N, 103°21'50.0"E; MUMNH MP00254. • 2 ♀♀, 2 ovigerous, Huai Yang, Nong Ya Sai, Wang Sam Mo; 16°57'22.9"N, 103°22'19.9"E; MUMNH MP00255. **Sakon Nakhon** • 2 ♀♀, 3 ♂♂, Nam Chan, Akat, Akat Amnuai; 17°35'46.1"N, 104°00'21.6"E; MUMNH MP00256. • 13 ♀♀, 4 ♂♂, Klong Lak, Chiang Khruea, Mueang Sakon Nakhon; 17°15'33.7"N, 104°07'00.1"E; MUMNH MP00257. • 2 ♀♀, 1 ♂, Nong Han, Tha Rae, Mueang Sakon Nakhon; 17°15'08.5"N, 104°09'32.0"E; MUMNH MP00258. • 3 ♀♀, 2 ♂♂, Klong Un, Khok Phu, Phu Phan; 17°00'22.1"N, 103°54'50.5"E; MUMNH MP00259. **Mukdahan** • 3 ♀♀, Klong Bang I, Nong Weang, Nikhom Kham Soi; 16°23'43.8"N, 104°34'33.7"E; MUMNH MP00260. **Kalasin** • 1 ovigerous, 4 ♂♂, Bueng Aram, Khlong Kham, Yang Talat; 16°24'21.8"N, 103°20'26.4"E; MUMNH MP00261. • 2 ♀♀, 3 ♂♂, Huai Sathot, Kham Bong, Huai Phueng; 16°41'32.6"N, 103°51'20.1"E; MUMNH MP00262. **Maha Sarakham** • 6 ♀♀, 1 ovigerous, 2 ♂♂, Chi River, Tha Tum, Mueang Maha Sarakham; 16°10'58.2"N, 103°27'19.9"E; MUMNH MP00263. • 1 ♀, Chi River, Koeng, Mueang Maha Sarakham; 16°13'01.1"N, 103°16'44.1"E; MUMNH MP00264. **Chaiyaphum** • 6 ♀♀, Huai I Muet, Khon San; 16°31'34.6"N, 101°39'28.0"E; MUMNH MP00265. • 3 ♀♀, 1 ovigerous, 2 ♂♂, Pha Iang Waterfall, Huai Ton, Mueang Chaiyaphum; 15°57'42.2"N, 101°54'17.8"E; MUMNH MP00266. • 1 ♀, Na Siao, Mueang Chaiyaphum; 15°55'05.1"N, 102°06'13.8"E; MUMNH MP00267. • 2 ♀♀, 2 ovigerous, Tat Ton, Na Siao, Mueang Chaiyaphum; MUMNH MP00268. **Roi Et** • 5 ♀♀, 4 ♂♂, Ban Nong Phue, Chaturaphak Phiman; 15°49'07.7"N, 103°30'36.9"E; MUMNH MP00269. **Ubon Ratchathani** • 2 ♀♀, 2 ovigerous, 2 ♂♂, Mekong River, Khong Chiam, Khong Chiam; 15°19'10.3"N, 105°29'53.2"E; MUMNH MP00270. • 4 ♀♀, Tung Lung, Nong Saeng Yai, Khong Chiam; 15°20'12.2"N, 105°24'02.6"E; MUMNH MP00271. **Si Sa Ket** • 1 ♀, Bueng Bun Local Market, Bueng Bun; 15°19'18.6"N, 104°03'01.2"E; MUMNH MP00272. • 2 ♀♀, 1 ovigerous, 2 ♂♂, Khayung, Thung Yai, Kantharalak 14°34'42.8"N, 104°38'47.5"E; MUMNH MP00273. **Nakhon Ratchasima** • 9 ♀♀, 1 ovigerous, 3 ♂♂, Lam Takhong, Mu Si, Pak Chong; 14°33'00.7"N, 101°27'34.1"E; MUMNH MP00274. • 2 ♀♀, 1 ♂, Sema, Sung Noen; 14°55'11.0"N, 101°47'53.5"E; MUMNH MP00275. **Lopburi** • 3 ♀♀, 1 ovigerous, 6 ♂♂, Lop Buri Local Market, Tha Sala, Mueang Lop Buri; 14°47'25.2"N, 100°40'27.7"E; MUMNH MP00276. **Nakhon Nayok** • 1 ♀, Khao Perm, Ban Na; 14°21'14.5"N, 101°05'06.9"E; MUMNH MP00277. **Chachoengsao** • 1 ♂, Nong Prue Mai Kaeo, Wang Yen, Plaeng Yao; 13°33'31.5"N, 101°17'44.0"E; MUMNH MP00278. **Sa Kaeo** • 3 ♀♀, 1 ovigerous, 3 ♂♂, Huai Yang, Thap Rat, Ta Phraya; 14°07'24.9"N, 102°40'03.6"E; MUMNH MP00279. **Chanthaburi** • 3 ♀♀, 5 ♂♂, Wang Kra Prae, Soi Dao; 12°58'18.9"N, 102°16'12.5"E; MUMNH MP00280. • 6 ♀♀, 5 ovigerous, 6 ♂♂, Koh Phasuk, Makham; 12°40'48.9"N, 102°12'08.6"E; MUMNH MP00281. • 10 ♀♀, 11 ♂♂, Phra Sathueng, Thap Chang, Soi Dao; 13°12'29.1"N, 102°10'07.0"E; MUMNH MP00282. • 2 ♀♀, 3 ♂♂, Klong I Ngaeo, Sung, Khlung; 12°27'13.8"N, 102°16'56.1"E; MUMNH MP00283. **Trat** • 1 ♀, Tha Sen, Khlong Yai; 12°06'53.3"N, 102°43'16.8"E; MUMNH MP00284. **Suphan Buri** • 6 ♀♀, 4 ovigerous, 12 ♂♂, Ban Chara Mai, Ban Kong, U Thong; 14°34'51.0"N, 99°52'04.7"E; MUMNH MP00285. • 1 ovigerous, 1 ♂, Kra Siao, Dan Chang; 14°50'01.9"N, 99°40'38.4"E; MUMNH MP00286. **Kanchanaburi** • 1 ♀, 1 ovigerous, 3 ♂♂, Huai Khayeng, Khayeng, Thong Pha Phum; 14°38'59.0"N, 98°34'31.3"E; MUMNH MP00287. • 1 ♀, Huai I Su, Nong Ri, Bo Phloi; 14°32'33.3"N, 99°22'41.4"E; MUMNH MP00288. • 3 ♀♀, 5 ♂♂, Taphoen, Lat Ya, Mueang Kanchanaburi; 14°08'14.0"N, 99°22'57.8"E; MUMNH MP00289. • 23 ♀♀, 3 ovigerous, 15 ♂♂, Huai Mae Pla Soi, Na Suan, Si Sawat; 14°34'03.4"N, 99°12'13.6"E; MUMNH MP00290. • 2 ♀♀, 5 ♂♂, Klong Phachi, Klon Do, Dan Makham Tia; 13°55'05.2"N, 99°22'59.3"E; MUMNH MP00291. **Ratchaburi** • 3 ♀♀, 1 ovigerous, 2 ♂♂, Khok Mu, Ta Nao Si, Suan Phueng; 13°28'29.5"N, 99°15'48.8"E; MUMNH MP00292. **Phetchaburi** • 2 ♀♀, Yang Chum, Klat Luang, Tha Yang; 12°46'55.7"N, 99°40'43.7"E; MUMNH MP00293. • 4 ♀♀, 2 ♂♂, Ban Yang Nam Klat Tai, Nong Ya Plong; 13°06'33.3"N, 99°43'22.8"E; MUMNH MP00294. **Prachuap Khiri Khan** • 2 ♂♂, Phongprasan, Bang Saphan; 11°12'53.1"N, 99°30'09.6"E; MUMNH MP00295. **Ranong** • 1 ovigerous, Bang Khun Paeng, Bang Phra Nuea, La-un; 10°04'06.0"N, 98°43'04.1"E; MUMNH MP00296. **Phang Nga** • 1 ♂, Phu Ta Jor, Le, Kapong; 8°46'04.7"N, 98°27'19.8"E; MUMNH MP00297. • 6 ♀♀, 1 ovigerous, 1 ♂, Thung Maphrao, Thai Mueang; 8°32'56.4"N, 98°19'23.4"E; MUMNH MP00298. **Phuket** • 1 ♂, Bang Pae, Pa Klok, Thalang; 8°02'17.8"N, 98°24'12.4"E; MUMNH MP00299. **Krabi** • 9 ovigerous, 1 ♂, Pak Nam, Mueang Krabi; 8°04'50.7"N, 98°55'07.3"E; MUMNH MP00300. **Songkhla** • 2 ♀♀, 1 ♂, Klong Na Thap, Chana; 7°01'20.1"N, 100°43'51.5"E; MUMNH MP00301.

##### Diagnosis.

Rostrum straight or proximal convex and distal margin gently upward. Rostrum length reaches beyond end of antennular peduncle and as long as scaphocerite. Rostral formula: 6–10/1–6 teeth including 1–3 distal teeth with small gap separate from rest. Carapace smooth. Epistome bilobed. First pereiopods reaching end of scaphocerite. Second pereiopods long and slender, similar in form and length, exceeding scaphocerite. Fingers covered with scattered setae, slightly shorter than palm. Translucent razor on cutting edge present anteriorly. Proximal quarter of cutting edges with one or two tiny teeth. Carpus cylindrical shape and articulation margin expanded. Carpus 1.5× longer than chela. Merus subcylindrical. Carpus 1.5× longer than merus. Third pereiopods long and slender, reaching end of scaphocerite. Dactylus curved distally with short setae. Propodus 2× longer than dactylus. Propodus with 4–8 pairs of spines distributed along its length and fine setae at articulation. Propodus 2× longer than carpus. Sixth and seventh thoracic sternites usually smooth. Eighth thoracic sternite with acute median process. First and second pleonal sternites with small median process. Third and fourth pleonal sternites smooth. Fifth pleonal sternite with triangular ridge. Uropodal diaeresis with inner movable spine shorter than outer angle.

**Figure 3.**
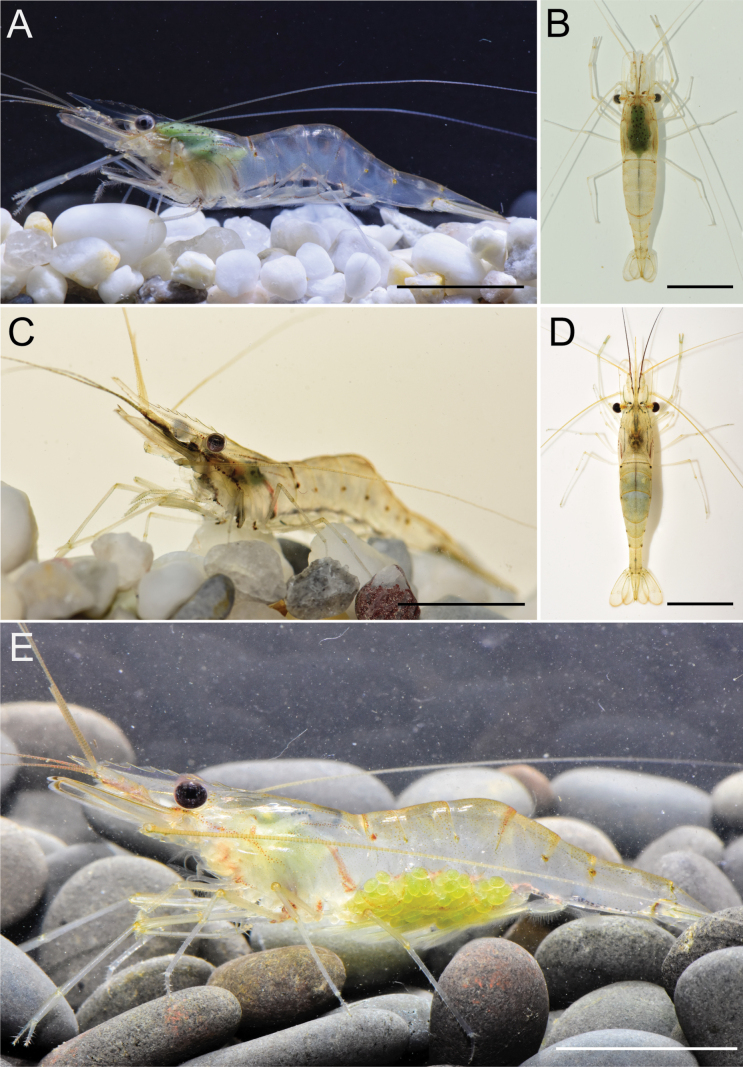
Living habit of specimens of three *Macrobrachium* species **A, B***M.lanchesteri* from Bang Khiat, Singhanakhon, Songkhla, Thailand **C, D***M.rostrolevatus* sp. nov. from Bueng Khong Long, Bueng Kan, Thailand **E***M.panhai* sp. nov. from Sri Nakarin Dam, Tha Kradan, Si Sawat, Kanchanaburi, Thailand. Scale bars: 1 cm.

##### Composite description.

**Figure 4.**
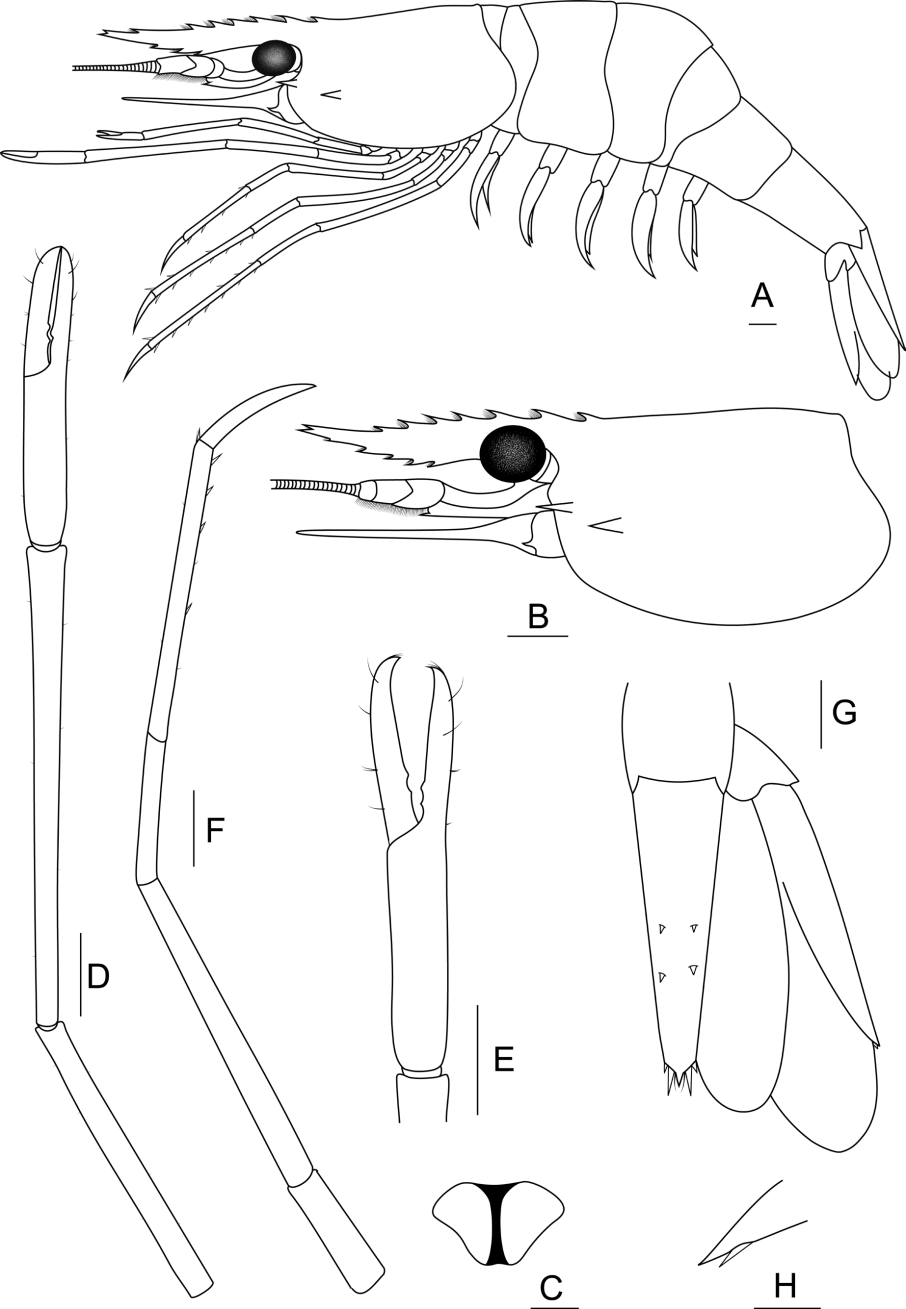
Morphological characteristics of *Macrobrachiumlanchesteri* (**A** female topotype MUMNH MP00218 **B–H** female topotype MUMNH MP00216.1) **A** lateral view **B** carapace **C** epistome **D** second pereiopod **E** teeth between fingers **F** third pereiopod **G** uropod and **H** movable spine at uropodal diaeresis. Scale bars: 1 mm.

***Rostrum*** (Fig. 4B). Straight or slightly convex proximally and upward distally. Rostrum length exceeding end of antennular peduncle and slightly shorter than scaphocerite. Dorsal margin with 6–10 teeth including 1–3 teeth distally with small gap from rest. Postorbital margin with one or two teeth, reaching to one-fourth of carapace length. Ventral margin with 1–6 teeth, starting from middle to distal margins. Short setae present between rostral teeth.

***Cephalon*** (Fig. 4B). Well-developed eye. Ocular beak without laterally expanded tip. Cornea longer and broader than stalk. Postantennular carapace margin rounded. Cornea osculum longer than stalk. Antennular peduncle longer than wide with fine setae, basal segment short, second segment shorter than third segment. Stylocerite projection sharp, reaching beyond basal segment. Antennal spine sharp situated below orbital margin. Hepatic spine slightly larger than antennal spine, positioned posteriorly and lower than antennal spine. Scaphocerite with straight margin, distolateral tooth sharp and not reaching end of lamella. Epistome bilobed (Fig. 4C). Branchiostegal suture starting from carapace margin to behind hepatic spine. Carapace surface smooth.

***First pereiopods.*** Long and slender, reaching end of scaphocerite. Fingers as long as palm, tips with fine setae. Series of setae present at anterior inner part of palm. Carpus slightly longer than merus. Distal articulation of carpus with series of fine setae. Ischium shorter than merus. Scattered setae present on all segments.

***Second pereiopods*** (Fig. 4D). Long and slender, similar in form and exceeding scaphocerite. Fingers subcylindrical covered with scattered setae. Palm 1.1–1.4× longer than fingers. Fingers with translucent razor edge present anteriorly and one or two tiny teeth on proximal quarter of cutting edges. Tip of fingers crossed and covered by fine setae (Fig. 4E). Carpus cylindrical shape and articulation margin expanded. Carpus 1.3–1.5× longer than chela. Merus subcylindrical. Carpus 1.1–2× longer than merus. Scattered short setae present on all segments.

***Third pereiopods*** (Fig. 4F). Long and slender, reaching end of scaphocerite. Dactylus short and curved distally. Propodus with 4–8 pairs of spines along inferior-lateral margin and fine setae at distal articulation, 2× longer than dactylus. Propodus 2× longer than carpus. Short setae present on all segments.

***Fourth and fifth pereiopods.*** Long and slender, exceeding scaphocerite. Propodus of fourth pereiopods with 5–10 pairs of spines distributed along its length, 2× longer than dactylus. Propodus slightly longer than merus. Ischium shorter than merus. Propodus with fine setae at distal articulation. Scattered short setae present on all segments. Propodus of fifth pereiopods with 7–13 pairs of spines distributed along its length and fine setae at distal articulation. Propodus 2× longer than carpus. Propodus as long as merus. Scattered short setae present on all segments.

***Thoracic sternum.*** Fourth and fifth thoracic sternites with transverse plate. Sixth and seventh thoracic sternites smooth. Eighth thoracic sternite with or without acute median process.

***Pleon.*** Smooth. All pleonal sternites with transverse ridge. First and second pleonal sternites usually with small median process. Third and fourth pleonal sternites smooth. Fifth pleonal sternite with triangular ridge. Preanal carina present, obtuse ridge developed without spine or setae. Ventral margin of pleural tergum with small setae.

***Telson*** (Fig. 4G). Tapered posteriorly, protruding point on middle margin with lateral spines and few fine setae. Inner spines longer than outer spines. Dorsal surface with two pairs of small spines similar in size.

***Uropods*** (Fig. 4G). Uropodal diaeresis with inner movable spine, usually shorter than outer angle. Exopods longer than endopods.

##### Remarks.

The specimen collected in this study generally agrees with the original description in Lanchester (1902), and a subsequent description of the lectotype provided by Chong and Khoo (1988). Previous studies reported that male specimens tended to display the sexual dimorphism with a large body size, tomentose fingers, and minute spinules on all segments (except fingers) of second pereiopods. In this study, only one large male specimen, collected from Loei Province, Thailand, exhibits this characteristic. Typically, both male and female specimens possess fine setae on fingers and scattered setae on surface of second pereiopods. Furthermore, this study also observed two variable characters occurring on the second pereiopods. Firstly, the proportional length and form of second pereiopods were found to be variable in specimens from Krabi population. Their second pereiopods are shown to be prominently long and robust, similar to those of *M.sintangense* (a common riverine species). The palm margin is laterally inflated and slightly shorter than fingers, and the chela slightly longer than the carpus. Additionally, Chong and Khoo (1988) reported the presence of two tiny teeth on the basal portion of cutting edges of fingers in *M.lanchesteri* as a diagnostic character. In this study, one or two tiny teeth were present on the cutting edges of fingers and vary among *M.lanchesteri* populations. Historically, *M.lanchesteri* was noted to resemble several other species including *M.idae*, *M.peguense* (see under remarks of *M.panhai* sp. nov.), *M.sankollii* Jalihal, Shenoy & Sankolli, 1988, *M.unikarnatakae* Jalihal, Shenoy & Sankolli, 1988, and *M.sintangense*. Further phylogenetic relationships and phylogenetic placement of aforementioned taxa should be tested to elucidate and verify their taxonomic identities.

*Macrobrachiumlanchesteri* has a wide distribution across mainland Southeast Asia and southern China. This species can live in various freshwater ecosystems by inhabiting aquatic vegetation in stagnant freshwater habitats such as ponds, lakes, and paddy fields.

#### ﻿ Macrobrachium panhai

Taxon classificationAnimaliaDecapodaPalaemonidae

Chaowvieng & Siriwut sp. nov.

FBC14CBB-8143-5781-A2ED-CECAF9DDB1B2

https://zoobank.org/42A0A555-C9CF-4DBF-B707-ED49A971F523

Figs 3E , 5

##### Material examined.

***Holotype*: Kanchanaburi** • Ovigerous ♀ from Sri Nakarin Dam, Tha Kradan, Si Sawat; 14°24'12.1"N, 99°07'24.7"E; CUMZ MP00302. ***Paratypes***: 7 ♀♀, 8 ovigerous, 6 ♂♂ from the same locality of holotype; MUMNH MP00303.

##### Additional material.

**Chiang Mai** • 6 ♀♀, 6 ovigerous, Nong Han, San Sai; 18°53'44.9"N, 99°01'05.7"E; MUMNH MP00304. • 1 ♀, Ping, Ki Lek, Mae Taeng; 19°04'37.0"N, 98°56'59.8"E; MUMNH MP00305. **Nan** • 1 ♀, Ban Na Bua, Nong Daeng, Mae Charim; 18°45'12.8"N, 101°00'39.7"E; MUMNH MP00306. **Uttaradit** • 1 ♂, Klong Tron, Ban Bueng Pra Kot, Thong Sang Khan; 17°35'39.1"N, 100°29'10.3"E; MUMNH MP00307. • 3 ♀♀, Klong Tron, Nam Khai, Nam Pat; 17°36'15.3"N, 100°32'15.3"E; MUMNH MP00308. **Buengkan** • 1 ♀, Klong Hi, Sang, Seka; 17°54'17.9"N, 103°57'12.0"E; MUMNH MP00351. **Nong Khai** • 2 ♀♀, Nam Suai, Song Hong, Mueang Nong Khai; 17°45'01.1"N, 102°51'00.5"E; MUMNH MP00309. **Ubon Ratchathani** • 1 ♀, 1 ovigerous, 2 ♂♂, Mekong River, Khong Chiam; 15°19'10.3"N, 105°29'53.2"E; MUMNH MP00310. • 1 ♀, 1 ovigerous, Sae Hua Maew Waterfall, Nong Saeng Yai, Khong Chiam; 15°20'12.2"N, 105°24'02.6"E; MUMNH MP00311. **Phitsanulok** • 6 ♀♀, 1 ♂, Ban Bo, Wang Nok Aen, Wang Thong; 16°51'13.6"N, 100°36'43.2"E; MUMNH MP00312. **Phetchabun** • 1 ♀, Ang Kep Nam Nam Khuen Nam Lang, Wang Hin, Wang Pong; 16°25'19.8"N, 100°48'48.2"E; MUMNH MP00313. **Nakhon Nayok** • 2 ovigerous, Ban Na, Pa Kha, Ban Na; 14°17'11.2"N, 101°04'13.7"E; MUMNH MP00314. **Suphan Buri** • 10 ♀♀, 8 ovigerous, 4 ♂♂, Ban Chara Mai, Ban Kong, U Thong; 14°34'51.0"N, 99°52'04.7"E; MUMNH MP00315. **Sa Kaeo** • 1 ovigerous, Huai Yang, Ta Phraya; 14°00'46.5"N, 102°38'37.0"E; MUMNH MP00316. **Chachoengsao** • 2 ♀♀, 3 ovigerous, Nong Prue Mai Kaeo, Wang Yen, Plaeng Yao; 13°33'31.5"N, 101°17'44.0"E; MUMNH MP00317. **Rayong** • 12 ovigerous, Khao Chuk, Kong Din, Klaeng; 12°51'32.0"N, 101°46'12.0"E; MUMNH MP00318. • 7 ♀♀, 11 ovigerous, 12 ♂♂, Koh Phasuk, Makham, Makham, Chanthaburi; MUMNH MP00319. **Trat** • 1 ♀, 2 ovigerous, Huai Raeng, Dan Chumphon, Bo Rai;12°23'48.4"N, 102°39'15.1"E; MUMNH MP00320. • 1 ♀, 2 ovigerous, Ang Kep Nam Dan Chumphon, Dan Chumphon, Bo Rai; 12°27'45.4"N, 102°38'24.6"E; MUMNH MP00321. **Phetburi** • 1 ♂, Klong Prachan, Yang Nam Klat Tai, Nong Ya Plong; 13°06'33.27"N, 99°43'22.75"E; MUMNH MP00322.

##### Diagnosis.

Rostrum straight proximally and slightly upward distally. Rostrum length reaching beyond end of antennular peduncle and exceeding the scaphocerite. Rostral formula: 8–12/3–6 teeth including two or three distal teeth with small gap separate from rest. Carapace smooth. Epistome bilobed. First pereiopods reaching end of scaphocerite. Second pereiopods thin and long, similar in form and equals in length, exceeding scaphocerite. Fingers covered with scattered setae, slightly shorter than palm. Translucent razor edge present anteriorly between fingers and no teeth on inner side of cutting edges. Carpus cylindrical shape and articulation margin expanded. Carpus 1.5× longer than chela. Merus subcylindrical. Carpus 1.5× longer than merus. Third pereiopods thin and long, reaching end of scaphocerite. Dactylus curved distally with short setae. Propodus 2× longer than dactylus. Propodus with three or four pairs of spines and fine setae present scarcely on articulation margin. Propodus 2× longer than carpus. Sixth to eighth thoracic sternites smooth. First and second pleonal sternites with small median process or smooth. Third and fourth pleonal sternites smooth. Fifth pleonal sternite with triangular ridge. Uropodal diaeresis with inner movable spine slightly longer than outer angle.

##### Composite description

**Figure 5.**
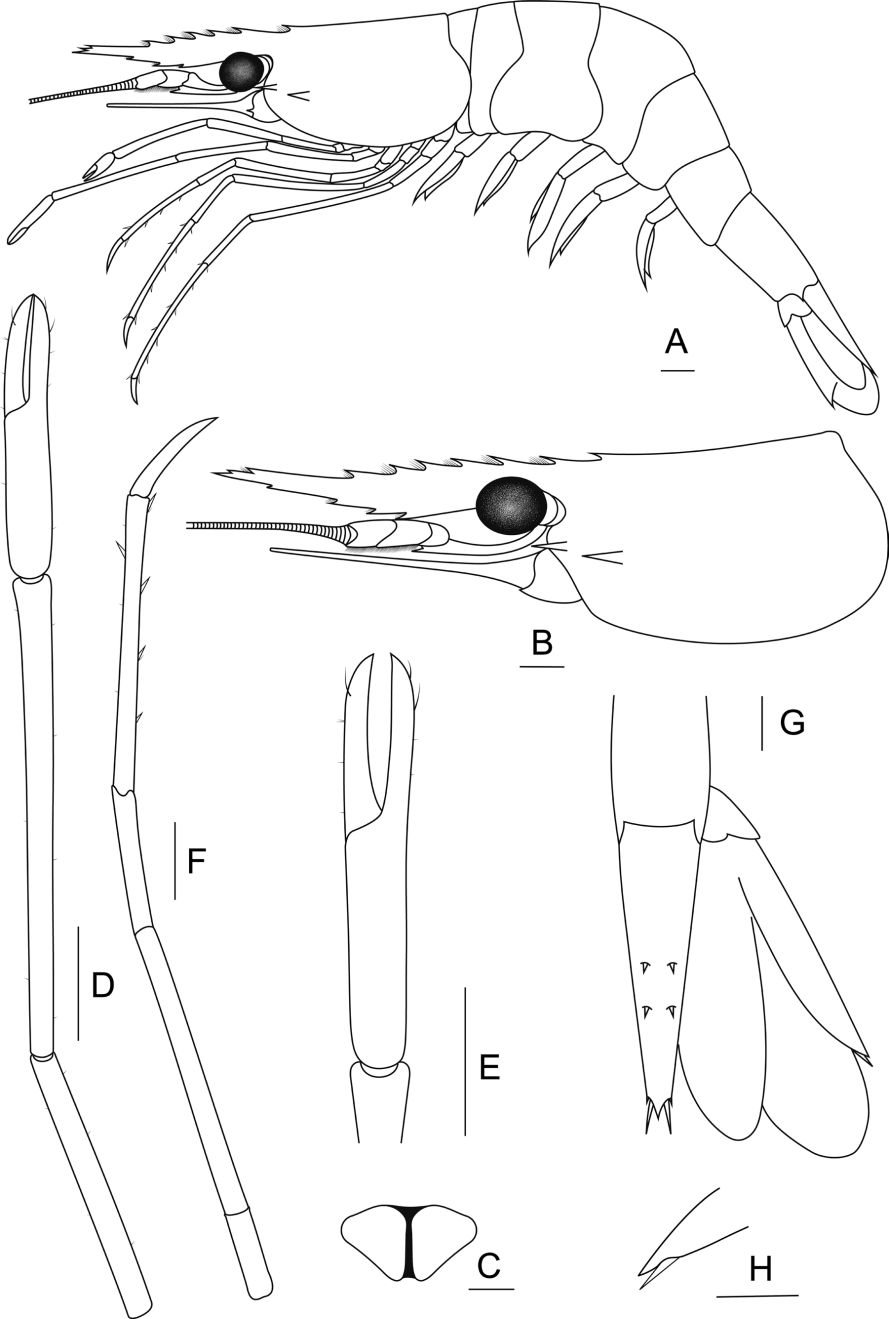
Morphological characteristics of *Macrobrachiumpanhai* sp. nov. (**A, F** ovigerous female paratype MUMNH MP00303 **B–E, G–H** ovigerous female holotype CUMZ MP00302) **A** lateral view **B** carapace **C** epistome **D** second pereiopod **E** teeth between fingers **F** third pereiopod **G** uropod and **H** movable spine at uropodal diaeresis. Scale bars: 1 mm.

**(holotype in parentheses). *Rostrum*** (Fig. 5B). Straight or proximal convex and slightly distal upward. Rostrum length exceeding end of antennular peduncle and slightly exceeding scaphocerite (rl 7.32 mm). Dorsal margin with 8–12 (10) teeth including two or three (3) teeth distally with small gap from rest. Postorbital margin with one or two (1) teeth, reaching one-third of carapace length. First dorsal tooth positioned slightly behind hepatic spine. Ventral margin with 3–6 (4) teeth, starting from middle to distal margin. Short setae present between rostral teeth.

***Cephalon*** (Fig. 5B). Eye well developed. Ocular beak without laterally expanded tip. Cornea longer and broader than stalk. Postantennular carapace margin rounded. Cornea osculum longer than stalk. Antennular peduncle longer than wide, with fine setae. Basal segment short, second segment shorter than third segment. Stylocerite projection sharp, reaching beyond basal segment. Antennal spine sharp, situated below orbital margin. Hepatic spine slightly larger than antennal spine, positioned posteriorly and lower than antennal spine. Scaphocerite with straight margin, distolateral tooth sharp and not reaching end of lamella. Epistome bilobed (Fig. 5C). Branchiostegal suture beginning at carapace margin to behind hepatic spine. Carapace surface smooth (cl 5.76 mm).

***First pereiopods.*** Thin and long, reaching end of scaphocerite. Fingers as long as palm, tips with fine setae. Series of setae present on anterior inner part of palm. Carpus slightly longer than merus. Distal articulation of carpus with series of fine setae. Ischium shorter than merus. Scattered setae present on all segments.

***Second pereiopods*** (Fig. 5D). Thin and long, similar in form and exceeding scaphocerite. Fingers subcylindrical covered with scattered setae. Palm 1.1–1.5× longer than fingers (Fin 1.06: Pal 1.39 mm). Fingers with translucent razor edges present anteriorly and cutting edge between fingers smooth. Tip of fingers crossed and covered by fine setae (Fig. 5E). Carpus cylindrical shape and articulation margin expanded. Carpus 1.2–2× longer than chela (Che 2.45: Car 4.22 mm). Merus subcylindrical. Carpus 1.2–1.7× longer than merus (Mer 2.85: Car 4.22 mm). Ischium as long as merus. Scattered short setae present on all segments.

***Third pereiopods*** (Fig. 5F). Thin and slender, reaching end of scaphocerite. Dactylus short and curved distally. Propodus 2× longer than dactylus. Propodus with three or four pairs of spines along inferior-lateral margin and fine setae at distal articulation, 2× longer than carpus. Ischium shorter than carpus. Scattered short setae present on all segments.

***Fourth and fifth pereiopods.*** Long and slender, exceeding scaphocerite. Propodus of fourth pereiopods with 3–6 (4) pairs of spines distributed along its length, 2.5× longer than dactylus. Propodus as long as merus. Ischium shorter than merus. Propodus with fine setae at distal articulation. Scattered short setae present on all segments. Propodus of fifth pereiopods with 4–8 pairs of spines distributed along its length and fine setae at distal articulation. Propodus 2.5× longer than carpus. Propodus as long as merus. Scattered short setae present on all segments.

***Thoracic sternum.*** Fourth and fifth thoracic sternites with moderately transverse plate without median process, and seventh thoracic sternite smooth. Eighth thoracic sternite usually smooth.

***Pleon.*** Smooth. All pleonal sternites with transverse ridges. First and second pleonal sternites with or without small median processes. Third and fourth pleonal sternites smooth. Fifth pleonal sternite with triangular ridge. Preanal carina present, obtuse ridge developed without spine or setae. Ventral margin of pleural tergum with small setae.

***Telson*** (Fig. 5G). Tapered posteriorly, protruding point on middle margin with lateral spines and few fine setae. Inner spines longer than outer spines. Dorsal surface with two pairs of small spines, similar in size.

***Uropods*** (Fig. 5G). Uropodal diaeresis with inner movable spine, as long as or slightly longer than outer angle. Exopods longer than endopods.

##### Etymology.

The specific name *panhai* is dedicated to Prof. Dr. Somsak Panha, a taxonomist from Faculty of Science, Chulalongkorn University, Thailand well known for his remarkable contributions and endorsement to the study of invertebrate fauna in Thailand.

##### Distribution.

This species is distributed in the Chao Phraya and Mekong River Basins, Thailand.

##### Remarks.

*Macrobrachiumpanhai* sp. nov. differs from *M.lanchesteri* s. str. due to having the rostral formula with 8–12/3–6 teeth (vs 6–10/1–6 teeth in *M.lanchesteri*). Movable spine at uropodal diaeresis is slightly longer than outer angle (vs movable spine is shorter in *M.lanchesteri*). Third propodus has 3–6 pairs of spines (vs 4–8 pairs of spines in *M.lanchesteri*). The teeth between fingers of second pereiopods are absent (vs 1 or 2 teeth on fixed and movable finger in *M.lanchesteri*). The ratio between rostrum and carapace length is 0.89–1.53 (vs 0.78–1.14 in *M.lanchesteri*) and the ratio between chela and carpus is 1.2–2.08 (vs 1.15–1.72 in *M.lanchesteri*). *M.panhai* sp. nov. occasionally co-exists with *M.lanchesteri* in the Chao Phraya and Mekong River Basins.

This new species also differs from *M.peguense* sensu Tiwari (1952) by processes of rostral formula 8–12/3–6 teeth (vs 6–9/2–4 teeth in *M.peguense*). Second pereiopods had palms shorter than half of carpus (vs palm slightly more than half of carpus in *M.peguense*). Propodus of third pereiopods are 2× longer than dactylus (vs 3 in *M.peguense*). Dorsal surface of telson is without depression (vs longitudinal depression in *M.peguense*). Movable spine at uropodal diaeresis is slightly longer than outer angle (vs movable spine is shorter in *M.peguense*). Cai and Ng (2002) also mentioned that the egg size can be used to distinguish *M.peguense* and *M.lanchesteri* group (1.15–1.5 × 1.6–2.1 mm and 0.6–0.7 × 0.8–1 mm, respectively). Currently, the distribution range of *M.peguense* was found only from Myanmar.

#### ﻿ Macrobrachium rostrolevatus

Taxon classificationAnimaliaDecapodaPalaemonidae

Chaowvieng & Siriwut sp. nov.

0B215504-B011-5117-BDFB-FA9D09A288EC

https://zoobank.org/58FE013B-93A1-43A0-9A96-7F45FBD759F9

Figs 3C, D , 6

##### Material examined.

***Holotype*: Bueng Kan** • Ovigerous ♀ from Bueng Khong Long; 17°59'59.1"N, 104°01'06.9"E; CUMZ MP00323. ***Paratypes***: 10 ♀♀, 13 ovigerous, 7 ♂♂ from the same locality of holotype; MUMNH MP00324.

##### Additional material.

**Nong Khai** • 3 ♀♀, 2 ♂♂, Nam Suai, Song Hong, Mueang Nong Khai; 17°45'01.1"N, 102°51'00.5"E; MUMNH MP00325. **Udon Thani** • 10 ♀♀, 4 ovigerous, 2 ♂♂, Si Charoen, Ban That, Phen; 17°42'47.7"N, 102°50'57.4"E; MUMNH MP00326. • 3 ♀♀, 1 ovigerous, Nam Khong, Thap Kung, Nong Saeng; 17°10'01.5"N, 102°46'03.2"E; MUMNH MP00327. **Loei** • 1 ♀, Tha Yang, Phu Kradueng; 16°53'38.3"N, 101°52'53.1"E; MUMNH MP00354. **Nakhon Phanom** • 2 ♀♀, 3 ♂♂, Klong Kam, Na Khu, Na Kae; 16°57'42.3"N, 104°31'33.2"E; MUMNH MP00328. • 1 ♀, Huai Saab, Sam Phong, Si Songkhram; 17°43'59.2"N, 104°09'19.9"E; MUMNH MP00329. **Sakon Nakhon** • 1 ♀, Nam Chan, Akat, Akat Amnuai; 17°35'46.1"N, 104°00'21.6"E; MUMNH MP00330. • 1 ♂, Klong Lak, Chiang Khruea, Mueang Sakon Nakhon; 17°15'33.7"N, 104°07'00.1"E; MUMNH MP00331. • 1 ♂, Klong Un, Khok Phu, Phu Phan; 17°00'22.1"N, 103°54'50.5"E; MUMNH MP00332. • 1 ♀, Tha Rae, Mueang Sakon Nakhon; 17°15'08.5"N, 104°09'32.0"E; MUMNH MP00352. **Kalasin** • 1 ♀, 2 ♂♂, Huai Sathot, Kham Bong, Huai Phueng; 16°41'32.6"N, 103°51'20.1"E; MUMNH MP00333. • 18 ♀♀, 2 ovigerous, 10 ♂♂, Bueng Aram, Khlong Kham, Yang Talat; 16°24'21.8"N, 103°20'26.4"E; MUMNH MP00334. **Khon Kaen** • 10 ♀♀, 2 ♂♂, Kong Kaeo Reservoir, Si Bun Rueang, Chonnabot; 16°05'47.2"N, 102°37'05.4"E; MUMNH MP00335. **Maha Sarakham** • 3 ♂♂, Ban Tha Tum, Mueang Maha Sarakham; 16°10'55.8"N, 103°27'10.6"E; MUMNH MP00336. **Yasothon** • 8 ♀♀, 11 ♂♂, Se Bai, Sawat, Loeng Nok Tha; 16°10'13.5"N, 104°32'21.1"E; MUMNH MP00337. • 11 ♀♀, 7 ovigerous, 12 ♂♂, Klong Wai, Fa Yat, Maha Chana Chai; 15°30'59.5"N, 104°15'12.3"E; MUMNH MP00338. **Si Sa Ket** • 13 ♀♀, 16 ovigerous, 2 ♂♂, Bueng Bun Local Market, Bueng Bun; 15°19'18.6"N, 104°03'01.2"E; MUMNH MP00339. • 2 ♀♀, Huai Khayung, Thung Yai, Kantharalak; 14°34'42.1"N, 104°38'48.1"E; MUMNH MP00340. • 1 ovigerous, Suk San, Phran, Khun Han; 14°35'27.7"N, 104°29'29.3"E; MUMNH MP00353. **Surin** • 3 ♀♀, Klong Thap Than, Yang Sawang, Rattanaburi; 15°16'55.4"N, 103°58'38.1"E; MUMNH MP00341. • 28 ♀♀, 9 ♂♂, Mun River, Tha Tum; 15°19'53.3"N, 103°38'34.9"E; MUMNH MP00342. • 12 ♀♀, 13 ♂♂, Mun River, Krapho, Tha Tum; 15°17'38.5"N, 103°30'42.4"E; MUMNH MP00343. • 3 ♀♀, Ban Kut Chum Saeng, Yawuek, Chumphon Buri; 15°18'57.6"N, 103°15'24.2"E; MUMNH MP00344. **Buri Ram** • 2 ovigerous, 1 ♂, Lam Chi, Non Charoen, Ban Kruat; 14°26'43.9"N, 103°12'55.8"E; MUMNH MP00345. **Nakhon Ratchasima** • 1 ♀, 7 ♂♂, Sathaet, Krabueang Nok, Mueang Yang; 15°27'35.2"N, 102°59'46.6"E; MUMNH MP00346. • 4 ♀♀, 3 ♂♂, Sema, Sung Noen; 14°55'11.0"N, 101°47'53.5"E; MUMNH MP00347. • 3 ♀♀, 2 ♂♂, Sawai Riang, Than Prasat, Non Sung; 15°16'13.3"N, 102°22'37.0"E; MUMNH MP00348. • 1 ♂, Lam Takhong, Mu Si, Pak Chong; 14°33'00.7"N, 101°27'34.1"E; MUMNH MP00349.

##### Diagnosis.

Rostrum long and thin, proximal half straight and uplifted distal half. Rostrum length reaching beyond end of antennular peduncle and prominently exceeding scaphocerite. Rostral formula: 6–11/4–9 teeth including 2–4 teeth distally with large gap from rest. Apical teeth usually present with trifid. Carapace smooth. Epistome bilobed. First pereiopods reaching end scaphocerite. Second pereiopods thin and long, similar in form and length, exceeding end of scaphocerite. Fingers covered with scattered setae with translucent razor edge present anteriorly between fingers and one tooth on proximal quarter of cutting edges. Palm 1.25× longer than fingers. Carpus cylindrical shape and articulation margin expanded. Carpus 1.5–2× longer than chela. Merus subcylindrical. Carpus 1.5× longer than merus. Third pereiopods thin and long, slightly exceeding scaphocerite. Dactylus curved distally with short setae. Propodus 2× longer than dactylus. Propodus with 3–6 pairs of spines distributed along its length and fine setae at its articulation. Propodus 2× longer than carpus. Sixth to eighth thoracic sternites smooth. First and second pleonal sternites with small median process. Third and fourth pleonal sternites smooth. Fifth pleonal sternite with triangular ridge. Uropodal diaeresis with inner movable spine slightly longer than outer angle.

##### Composite description

**Figure 6.**
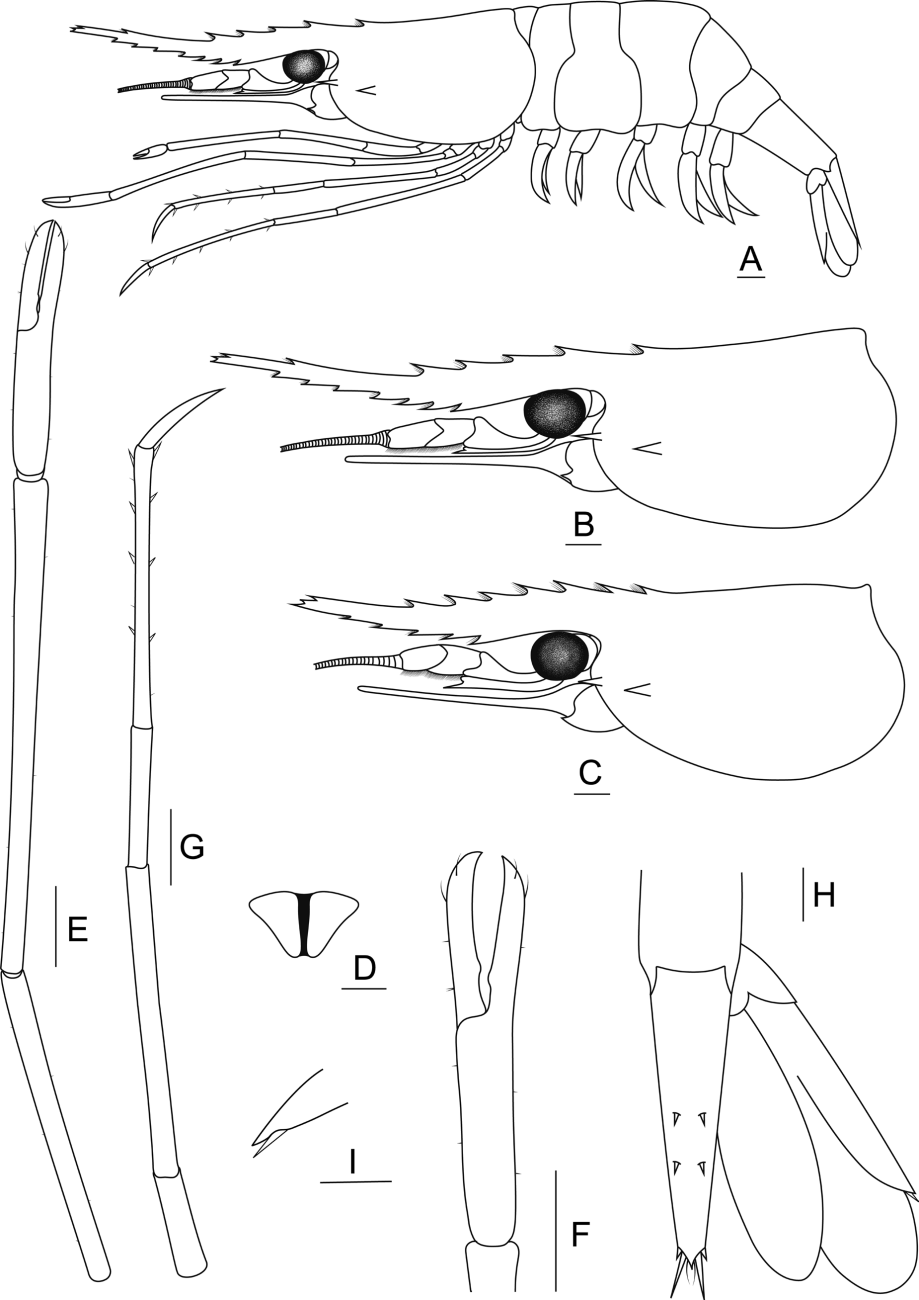
Morphological characteristics of *Macrobrachiumrostrolevatus* sp. nov. (**A, B, D–I** ovigerous female holotype CUMZ MP00323 **C** ovigerous female specimen MUMNH MP00338.1) **A** lateral view **B** carapace **C** rostral variation **D** epistome **E** second pereiopod **F** teeth between fingers **G** third pereiopod **H** uropod and **I** movable spine at uropodal diaeresis. Scale bars: 1 mm.

**(holotype in parenthesis). *Rostrum*** (Fig. 6B). Tapered and long, proximal half of rostrum straight and uplifted distally. Rostrum length exceeding end of antennular peduncle and distinctly exceeding scaphocerite (rl 10.34 mm). Dorsal margin with 6–11 (9) teeth including 2–4 (4) teeth distally separated from rest. Apical teeth usually present with trifid. Postorbital margin with 1 or 2 (1) teeth reaching one-third of carapace length. First dorsal tooth positioned slightly behind hepatic spine. Ventral margin with 4–9 (7) teeth, starting from middle to distal margin. Short setae present between rostral teeth.

***Cephalon*** (Fig. 6B). Eye well developed; ocular beak without laterally expanded tip. Cornea longer and broader than stalk. Postantennular carapace margin rounded. Cornea osculum longer than stalk. Antennular peduncle longer than wide, with fine setae. Basal segment short, second segment being shorter than third segment. Stylocerite projection sharp, reaching beyond basal segment. Antennal spine sharp, situated below orbital margin. Hepatic spine slightly larger than antennal spine, positioned posteriorly and lower than antennal spine. Scaphocerite with straight margin, distolateral tooth sharp and not reaching end of lamella. Epistome bilobed (Fig. 6D). Branchiostegal suture starting from carapace margin to behind hepatic spine. Carapace surface smooth (cl 7.14 mm).

***First pereiopods.*** Long and slender, reaching end of scaphocerite. Fingers as long as palm, tips with fine setae. Series of setae present at anterior inner part of palm. Carpus slightly longer than merus. Distal articulation of carpus with series of fine setae. Ischium shorter than merus. Scattered setae present on all segments.

***Second pereiopods*** (Fig. 6E). Long and slender, similar in form and distinctly exceeding the scaphocerite. Fingers subcylindrical covered with scattered setae. Palm 1.2–1.7× longer than fingers (Fin 1.47: Pal 1.84 mm). Fingers with translucent razor edge present anteriorly and one tooth on cutting edges. Tip of fingers crossed and covered by fine setae (Fig. 6F). Carpus cylindrical shape and articulation margin expanded. Carpus 1.5–2.0× longer than chela (Che 3.31: Car 6.19 mm). Merus subcylindrical. Carpus 1.3–1.6× longer than merus (Mer 3.99: Car 6.19 mm). Ischium as long as merus. Scattered short setae present on all segments.

***Third pereiopods*** (Fig. 6G). Long and slender, slightly exceeding scaphocerite. Dactylus curved distally with short setae. Propodus 2× longer than dactylus (Dac 1.19: Pro 3.94 mm). Propodus with 3–6 (4) pairs of spines along inferior-lateral margin and fine setae at distal articulation, 2× longer than carpus (Car 1.83: Pro 3.94 mm). Ischium shorter than carpus. Scattered short setae present on all segments.

***Fourth and fifth pereiopods.*** Long and slender, exceeding scaphocerite. Propodus of fourth pereiopods with 4–7 (5) pairs of spines distributed along its length, 2× longer than dactylus. Propodus slightly shorter than merus. Ischium shorter than merus. Propodus with fine setae at distal articulation. Scattered short setae present on all segments. Propodus of fifth pereiopods with 4–10 pairs of spines (holotype damaged) distributed along its length and fine setae at distal articulation. Propodus 2.5× longer than carpus. Propodus as long as merus. Scattered short setae present on all segments.

***Thoracic sternum.*** Fourth and fifth thoracic sternites with moderately transverse plate. Sixth to eighth thoracic sternites usually smooth.

***Pleon.*** Smooth. All pleonal sternites with transverse ridge. First and second pleonal sternites with or without median process. Third and fourth pleonal sternites smooth. Fifth sternite with triangular ridge. Preanal carina present, obtuse ridge developed without spine or setae. Ventral margin of pleural tergum with small setae.

***Telson*** (Fig. 6H). Tapered posteriorly, protruding point on middle margin with lateral spines and few fine setae. Inner spines longer than outer spines. Dorsal surface with two pair of small spines, similar in size.

***Uropods*** (Fig. 6H). Uropodal diaeresis with inner movable spine, as long as or slightly longer than outer angle. Exopods longer than endopods.

##### Etymology.

The specific epithet *rostrolevatus* is from the Latin compound words *rostro*, for rostrum, and *levatus*, referring to lifted.

##### Distribution.

This species is distributed in freshwater basins of Khorat Plateau, Northeast Thailand.

##### Remarks.

*Macrobrachiumrostrolevatus* sp. nov. differs from *M.lanchesteri* s. str. based on the presence of single tooth on movable and fixed fingers of second pereiopods (vs 1 or 2 teeth on movable and fixed fingers in *M.lanchesteri*), movable spine at uropodal diaeresis slightly longer than the outer angle (vs shorter than outer angle in *M.lanchesteri*), and the presence of 3–6 pairs of spines on propodus of third pereiopods (vs 4–8 pairs of spines in *M.lanchesteri*). This new species also differs from *M.villosimanus* sensu Tiwari (1949) and *M.rosenbergii* sensu De Man (1879) by having 6–11/4–9 rostral teeth (vs 12–14/7–10 rostral teeth in *M.villosimanus*; 9–13/10–15 rostral teeth in *M.rosenbergii*). The second pereiopods are smooth and covered with fine setae (vs spinules in entire cheliped, movable finger densely pubescent and fixed finger sparsely pubescent in *M.villosimanus*; coarse velvet hairs on movable finger except its tip, and fixed finger covered with numerous short spines in *M.rosenbergii*). Moreover, this new species differs from *M.lamarrei* sensu H. Milne Edwards (1837) by processes 6–11/4–9 rostral teeth (vs 6–11/5–9 rostral teeth in *M.lamarrei*). The movable spine at uropodal diaeresis is slightly longer than outer angle (vs without movable spine in *M.lamarrei*). Further description of *M.lamarrei* was provided by Cai and Ng (2002). In addition, *M.rostrolevatus* sp. nov. is present only in freshwater basins on the Khorat Plateau, and lives in various habitats such as lakes, ponds, and river whereas *M.villosimanus*, *M.rosenbergii* and *M.lamarrei* typically inhabit brackish water territory.

*Macrobrachiumrostrolevatus* sp. nov. exhibits phenotypic plasticity in rostral shape. The population in a lentic habitat such as a pond, paddy field and lake have an upcurved on distal half of rostrum. On the other hand, some populations have slightly convex at basal and upturned distally with a smaller gap between distal and proximal teeth of rostrum (Fig. 6C). The taxonomic discrimination based on rostrum form in genus *Macrobrachium* is cautioned due to controversial situation found in this study. Additionally, the uncertain identity found from Thai *Macrobrachium* specimens was also mentioned in previous records. For example, Naiyanetr (1998) reported *M.palaemonoides* Holthuis, 1950 [= *Tenuipediumpalaemonoides* in Wowor and Ng (2010)] from Surin Province. Re-examination of the collection by Cai et al. (2004) placed those specimens back under a typical variation of *M.lanchesteri* and mentioned that *T.palaemonoides* s. str. was known only from the original type locality in the west coast of Sumatra. The unique characteristics of *T.palaemonoides* are shown to be distinct from *Macrobrachium* such as its long branchiostegal groove, the second pereiopod is as wide as first pereiopod and the fourth and fifth pereiopods are longer than the second pereiopod.

### ﻿Key to species of *M.lanchesteri* and closely related species in Thailand

**Table:** 

1	Rostrum upturned distally and exceeding the scaphocerite by one-third its length	**2**
–	Rostrum straight and as long as scaphocerite	**4**
2	Fingers of second pereiopods without pubescence, fifth pereiopods with spiniform setae	***M.rostrolevatus* sp. nov.**
–	Finger of second pereiopods with pubescence, fifth pereiopods with extremely spiniform setae	**3**
3	Rostrum with 7–10 ventral teeth, carpus subequal to chela, densely pubescent on movable finger and sparsely pubescent for fixed finger	***M.villosimanus***
–	Rostrum with 10–14 ventral teeth, carpus longer than palm, coarse velvet hairs on movable finger except the tip	***M.rosenbergii***
4	Second pereiopods more robust and longer than body length, rostrum with 10–14 dorsal teeth	***M.sintangense* group**
–	Second pereiopods slenderly without spine and shorter than body length, rostrum with 6–12 dorsal teeth	**5**
5	Fingers with two tiny teeth on cutting edges, movable spine at uropodal diaeresis shorter than outer angle	***M.lanchesteri* s. str.**
–	Fingers without teeth on cutting edges, movable spine at uropodal diaeresis longer than outer angle	***M.panhai* sp. nov.**

## ﻿Discussion

Morphological and genetic analyses revealed three distinct lineages (prior assumption as geographical variation of *M.lanchesteri*), which are recognised herein as *M.lanchesteri* s. str., *M.panhai* sp. nov., and *M.rostrolevatus* sp. nov. Previously, the taxonomic identity of *M.lanchesteri* s. l. was investigated based on the morphological examination of and reinvestigation of type specimens (Lanchester 1902; Chong and Khoo 1988). In this study, the clarification of species boundaries and phylogenetic positions were supplemented by molecular analyses. The phylogenetic position of *M.lanchesteri* is closely related to *M.rosenbergii*, although some morphological characteristics might appear similar to the *M.sintangense* species group. Current observation noted that a juvenile of *M.sintangense* and *M.lanchesteri* were morphologically overlapping. Ecologically, they commonly co-exist in several habitats such as riverbanks and lentic reservoirs in mainland Southeast Asia. Their life histories were supposedly influenced by a convergent evolutionary mechanism (Wowor et al. 2009), the same example as noted in other species with abbreviated larval development (ALD) such as *Macrobrachium* species: *M.platycheles* Ou & Yeo, 1995, *M.sundaicum* (Heller, 1862), and *M.malayanum* (Roux, 1935) (Murphy and Austin 2005). The independent lineages of ALD species were hypothesised as evidence of multiple invasions of marine ancestors (Liu et al. 2007; Murphy and Austin 2005; Wowor et al. 2009). To elucidate the effect of environmental conditions and feeding preferences altering morphological characteristics among coexisting species, comprehensive materials along an environmental gradient could be investigated. Additionally, *M.rosenbergii* showed distinctiveness in both morphological characters and a reproductive strategy different from *M.lanchesteri*. The life cycle of *M.lanchesteri* is completed typically in freshwater as opposed to *M.rosenbergii*, which had larval development and egg hatching occurring in brackish water. The close phylogenetic relationship between *M.lanchesteri* and *M.rosenbergii* seem to potentially derive from a common ancestor through evolutionary divergence processes.

The evidence of genetic divergence and composition differences in Thai invertebrate population are often documented between the lower and upper Isthmus of Kra regions. This evidence was sparsely seen in *M.lanchesteri* s. str. The same patterns of genetic divergence correlated to subregional populations were also detected in the widespread *M.spinipes* (Schenkel, 1902). This species shows a wide distribution range in the Indo-Australasian region due to a historical event during the last glacial maximum (De Bruyn and Mather 2007; Ng and Wowor 2011). Currently, the geographical distribution of *M.lanchesteri* in Southeast Asia seems to possibly include the introduction by human activities, particularly from local fishery-related activities such as in Sabah and Brunei Darussalam (Ng 1994; Wowor and Choy 2001). Thai *M.lanchesteri* s. str. failed to show a strong subregional pattern despite widespread distribution records, and a similar pattern was also observed in some freshwater gastropods collected from different parts of Thailand (Saijuntha et al. 2021). This might be the consequence of the commercial trade of aquatic plants in Thailand that accidentally introduced freshwater gastropods throughout the area. Contrastingly, *M.rostrolevatus* sp. nov. has a narrow distribution range and a dense population specifically found in the sub-basins of the Songkhram, Chi, and Mun rivers on the Khorat Plateau. However, a comprehensive survey of the adjacent sub-basins along the Lower Mekong River Basin should be implemented to affirm its geographic range.

*Macrobrachium* prawns exhibit a vast variation of morphological characters, with several species demonstrating sexual dimorphism and morphological plasticity (Holthuis 1950; Dimmock et al. 2004; Short 2004). These phenomena increased the uncertainty of species boundaries and the complication of taxonomic discrimination criteria for various *Macrobrachium* species groups. Recent studies have employed tools, including molecular identification using mitochondrial gene datasets, to clarify and resolve taxonomically ambiguous situations (Liu et al. 2007; Carvalho et al. 2013; Castelin et al. 2017; Rossi et al. 2020; Saengphan et al. 2021). In this study, the mitochondrial genes 16S and COI showed potential to be useful for taxonomic clarification between closely related taxa and revealed the existence of cryptic species, as in the cases of *M.panhai* sp. nov. and *M.lanchesteri* s. str. Although *M.panhai* sp. nov. shares morphological characteristics with *M.lanchesteri*, genetic differentiation falls within the delimitation gap suggested by Siriwut et al. (2021). For this reason, the delimitation threshold based on inter- and intraspecific variations of *Macrobrachium* species would be considered an additional tool for cryptic fauna exploration and delineation of morphologically ambiguous groups of *Macrobrachium* prawns.

*Macrobrachiumrostrolevatus* sp. nov. has different forms of rostrum that appear to be associated with habitat preference. The long and upcurved rostrum is prevalent in lentic habitats i.e., ponds and lakes, whereas the shorter and straight rostrum is dominant in lotic habitats like river tributaries. This rostral shape variability may indicate phenotypic plasticity, similar to observations in *M.australe* (Guérin-Méneville, 1838) and members of the genus *Caridina* H. Milne Edwards, 1837, where rostral shape is influenced by water current speed. In an area with fast-flowing current, the long rostrum can be more fragile and impede movement whereas the shorter, more robust, and straight rostrum might better resist the strong water current (Zimmermann et al. 2011; Mazancourt et al. 2017). Moreover, the variation in morphological traits influenced by environment was also found in *M.australiensis* Holthuis, 1950, an endemic Australian freshwater prawn and *M.nipponense* (De Haan, 1849), a widespread species in Taiwan (Dimmock et al. 2004; Chen et al. 2015). This study provided additional evidence that the diagnostic characters of *Macrobrachium* can be influenced by the environment. Therefore, morphological identification alone should be implemented carefully, especially for species with high morphological variability (Liu et al. 2007; Siriwut et al. 2020). The integration of other molecular markers such as nuclear markers and morphometric analysis could be used to further enhance the accuracy of taxonomic identification and phylogenetic relationships of *Macrobrachium* in the future.

## Supplementary Material

XML Treatment for Macrobrachium lanchesteri

XML Treatment for Macrobrachium panhai

XML Treatment for Macrobrachium rostrolevatus

## ﻿Additional information

### Author ORCIDs

Chirasak Sutcharit https://orcid.org/0000-0001-7670-9540

Ratmanee Chanabun https://orcid.org/0000-0003-4584-2773

Ruttapon Srisonchai https://orcid.org/0000-0002-7142-0999

Warut Siriwut https://orcid.org/0000-0001-6760-9724

### Data availability

All of the data that support the findings of this study are available in the main text.
